# Effects of Schlemm’s Canal Expansion: Biomechanics and MIGS Implications

**DOI:** 10.3390/life11020176

**Published:** 2021-02-23

**Authors:** Chen Xin, Shaozhen Song, Ningli Wang, Ruikang Wang, Murray Johnstone

**Affiliations:** 1 Beijing Tongren Eye Center, Beijing Institute of Ophthalmology, Beijing Tongren Hospital, Capital Medical University, Beijing 100730, China or xinchen0322@ccmu.edu.cn (C.X.); or wningli@ccmu.edu.cn (N.W.); 2 Department of Bioengineering, University of Washington, Seattle, Washington, WA 98195, USA; szsong@uw.edu (S.S.); wangrk@uw.edu (R.W.); 3 Department of Ophthalmology, University of Washington, Seattle, Washington, WA 98195, USA

**Keywords:** trabecular meshwork, schlemm’s canal, biomechanics, optical coherence tomography

## Abstract

Objective: To evaluate the change of biomechanical properties of the trabecular meshwork (TM) and configuration of collector channels (CC) by high-resolution optical coherence tomography (HR-OCT) induced by Schlemm’s canal (SC) dilation. Methods: The anterior segments of two human eyes were divided into four quadrants. One end of a specially designed cannula was placed in SC and the other end connected to a perfusion reservoir. HR-OCT provided three-dimensional (3D) volumetric and two-dimensional (2D) cross-sectional imaging permitting assessment of the biomechanical properties of the TM. A large fluid bolus was introduced into SC. Same-sample, pre and post deformation and disruption of SC and CC lumen areas were analyzed. Results: Morphologic 3D reconstructions documented pressure-dependent changes in lumen dimension of SC, CC, and circumferential intrascleral channels. 2D imaging established volumetric stress-strain curves (elastance curves) of the TM in quadrants. The curves of TM elastance shift to the right with an increase in pressure-dependent steady-state SC area. After a bolus disruption, the SC area increased, while the CC area decreased. Conclusion: Our experimental setup permits the study of the biomechanical properties of TM by examining elastance, which differs segmentally and is altered by mechanical expansion of SC by a fluid bolus. The study may shed light on mechanisms of intraocular pressure control of some glaucoma surgery.

## 1. Introduction

Glaucoma is a leading cause of irreversible blindness, and primary open-angle glaucoma (POAG) is the most common type. The cause of glaucoma remains an enigma, but stiffening and loss of pulse-dependent bulk motion of the trabecular meshwork (TM) tissues are implicated as an important factor. For example, TM motion that permits Schlemm’s canal (SC) to fill with blood with pressure gradient reversal becomes abnormal and eventually stops as glaucoma progresses [1,2,3]. In addition, pulsatile aqueous flow from SC to the episcleral veins slows and eventually stops in glaucoma but is restored by drugs that reduce intraocular pressure (IOP) [4].

SC functions as a compressible chamber. Its morphology and behavior suggests that it is a component of a lymphatic-like pump that regulates IOP [5]. Many studies and multiple lines of evidence document that flow to the aqueous veins is pulsatile and that the origin of the aqueous inducing pulsatile flow is SC and the distal outflow channels [6,7]. Especially compelling evidence is that pulsatile aqueous in the aqueous veins increases when access to the episcleral veins is blocked in normal veins [8,9]. The pulsatile flow requires the presence of TM motion since it is the compressible tissue capable of changing SC dimensions to induce pulsations.

Optical coherence tomography (OCT) imaging is shedding new light on TM motion, providing evidence that TM motion becomes abnormal in glaucoma. Imaging in humans demonstrates that distention of the TM into SC is dependent on IOP [10]. OCT studies demonstrate both in the laboratory and in patients that the TM undergoes cyclic motion induced by the ocular pulse [11,12]. Reduced TM motion occurs experimentally in response to a decrease in the ocular pulse that is associated with the closure of SC [11]. These OCT studies indicate that biomechanical properties define pressure-dependent TM distention into SC and determine oscillatory responses to the ocular pulse in normal subjects.

Recent evidence suggests that OCT can characterize abnormal responses in glaucoma [13]. Recent high-resolution OCT (HR-OCT) studies demonstrate synchronous real-time changes in the lumen dimensions of SC and collector channel (CC) in response to pressure variation [14,15,16]. These OCT studies, as well as scanning electron microscopy and micro-CT, identify comparable structures in the TM pathways [17,18,19]. Our current study extends information related to the biomechanical behavior of the outflow system by further exploring tissue elastance. Elastance determines the ability of the TM tissues of SC inner wall to appropriately distend and recoil. Optimization of elastance is essential to control normal IOP, a property that becomes abnormal in glaucoma.

Medications and laser procedures are often ineffective in controlling IOP, so many patients have required interventional surgery. Trabeculectomy has for many years been the most common interventional surgery, but the risk-benefit ratio makes its use problematic, except in those needing very low pressures. Micro-invasive glaucoma surgery (MIGS) procedures aimed at restoring flow through the aqueous outflow pathway are attractive because they generally attain pressures in the mid-teens and have a good safety profile. Our pilot effort in this report explores the changes in biomechanical responses resulting from MIGS-like dilation of SC. Canal expansion is done with procedures that use viscoelastics to dilate SC, such as external or ab interno canaloplasty (AbiC) or instrumentation of the canal with sutures or cannulas.

Previous experimental studies have cannulated SC and dilated it with viscoelastic or the passage of sutures with dimensions comparable to cannulas [20]. Assessment of effects has been done with light and scanning electron microscopy. Cannulation by sutures the size of cannulas disrupts the SC inner wall, compresses the external wall, and tears away endothelial tubes connecting the TM to hinged collagen flaps at CC entrances [21]. Viscoelastic also overdilates SC distal at the site of infusion cannula insertion. The biomechanical effects of disruption of SC tissues have not previously been amenable to study. Of special interest are pressure-dependent biomechanical responses after disruption of connections between the TM and hinged flaps at SC ostia.

Our goal in this pilot study is to explore the biomechanical properties of the outflow system resulting from changes in SC pressure. Our first aim is to explore changes in structural features and relationships of SC, CC, and distal pathways using three dimensional (3D) volumetric imaging. A second aim is to assess the mechanical properties of the TM through the quantitation of stress-strain relationships. A final aim is to explore the changes in biomechanical behavior of the outflow system after disruption of canal structures by mechanisms that simulate canal-based MIGS procedures.

## 2. Materials and Methods

### 2.1. Tissue Preparation

Normal human eyes used in this study were provided by the Sightlife™ eye bank within 24 h after the donor’s death. The donors’ ages were 53 (Eyes #1 and #2) and 67 (Eye #3). Both were Caucasian with no history of eye disease. The 12 o’clock limbal position was marked, and the eye hemisected followed by removal of the lens and iris. We then divided the anterior segment into four quadrants (superior temporal, ST; inferior temporal, IT; superior nasal, SN; and inferior nasal, IN). The wedge-shaped limbal quadrant with the TM facing upward toward the OCT beam was immersed in Hanks balanced salt solution in the Petrie dish. The cornea and sclera of the quadrant were affixed to the dish using pins that pierced an underlying layer of silicon preplaced within the dish [15].

### 2.2. Experimental Protocol

The perfusion system consisted of two perfusion reservoirs, a perfusion pump, and a three-way switch. One end of a laboratory-fashioned 150 µm steeply tapered cannula tip was inserted into the cut end of SC and connected to PE 60 tubing leading to a three-way switch. Quantitative measurement of pressure-dependent SC dimensions was achieved as follows. In each quadrant, the SC cannula led to a single reservoir to provide controlled steady-state hydrostatic pressures of 0, 5, 10, 20, 30, and 50 mmHg. At each steady-state condition, both two dimensional (2D) cross-sectional images and 3D volumetric images were acquired with the OCT system.

Using the same quadrants as in the steady-state experiment, we next simulated expansion of SC as is done with viscoelastics. We used the three-way switch to connect the SC cannula to a perfusion pump. Perfusion pump parameters were set as follows: speed, 10 mL/min; volume, 125 µL; speed 20 mL/min, volume 125 µL; speed, 30 mL/min, 125 µL; speed 30 mL/min, volume 250 µL. After the introduction of the BSS bolus, steady-state measurements were again made to determine whether alterations in biomechanical properties of the tissues had occurred.

### 2.3. Imaging System and Scanning Procedures

The HR-OCT system consists of a light source with a central wavelength of 1340 nm and a half-bandwidth of 110 nm, a 1024-pixel built-in high-speed spectrometer, and a 92 kHz A-line InGaAs linear scan camera. The output power of the OCT system is 2.5 mW with ~105 dB energy at the spot focus. The scan rate is 200 frames per second with an axial resolution of 7.2 µm in tissue (5 µm in the air), transverse resolution of 5 µm, imaging depth of 2.2 mm, and a focus range of 0.5 mm. During imaging, a glass sheet covered the fluid surface to reduce the impact of surface fluctuations on imaging [15].

The range of 3D imaging was 2 × 2 × 3 mm^3^, which was composed of 512 B-scan frames with each frame composed of 360 equally spaced A-lines. With the 3D image acquisition mode, the 3D structure of SC, CC, and ISCC could be clearly identified. The 2D acquisition model was used to show the variation of the profile of the same-sample SC section under conditions of changing perfusion pressure and were acquired over 7 s.

### 2.4. Data Analysis

Amira software was used to reconstruct the 3D volumetric images of SC. The optimal angle was selected to observe the structural relationships of SC, CC, and intra-scleral collector channel (ISCC) adjacent to SC. For analysis of the 2D images, the boundary between SC and CC was semi-automatically delineated. ImageJ software then provided delineation of the SC and CC area [6]. The total area of SC within a 2 mm length was automatically calculated from the 3D images. Stress-strain curves were developed to assess the biomechanical characteristics of the TM that determined its response to changing SC pressure. The analysis compared curves before and after a fluid bolus that disrupts TM-CC connections.

## 3. Results

### 3.1. D Volumetric Imaging

**Figure 1:**
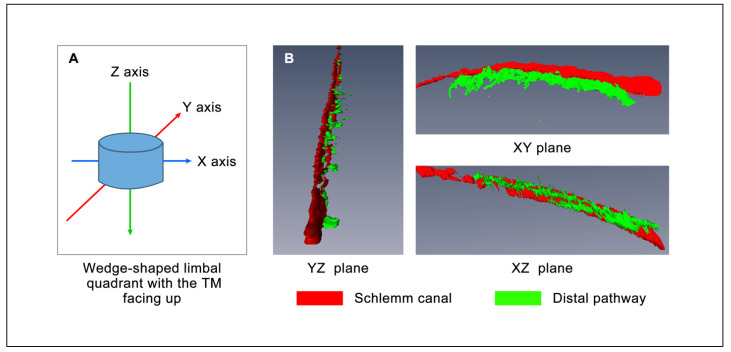
3D-OCT images of SC and its distal aqueous outflow pathway with a pressure of 30 mmHg. (**A**) Model of the scanning azimuth. (**B**) The structure of SC and its distal aqueous outflow pathways from different angles. D-OCT: three-dimensional-optical coherence tomography, SC: Schlemm’s canal.

HR-OCT volumetric images provide a 3D view demonstrating relationships between SC, CC, and ISCC (Figure 1 Eye #1 superior temporal quadrant while maintaining a steady-state pressure of 30 mm Hg). The site of cannula insertion is visible as a funnel-shaped area at the right edge of the SC image. SC dimensions gradually decrease as distances distal to the site of the cannula insertion increase. The XZ plane in Figure 1 demonstrates the CC departing the canal in a relatively narrow plane, rather than arising from multiple surfaces of SC lumen. The XY plane captures an orthogonal view of CC entrances further emphasizing the uniform plane of departure from SC. In the XY plane, circumferential ISCC have an orientation parallel to SC, have a distended configuration, and encompass an area similar in size to that of SC.

**Figure 2:**
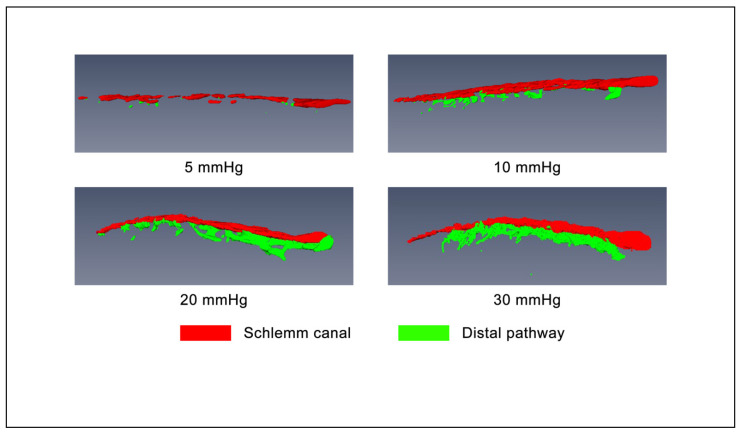
Structural connections between Schlemm’s canal and its distal pathways at different perfusion pressures oriented in the X-Y plane.

Figure 2 panels maintain the orientation of the XY plane of Figure 1 and demonstrate progressive enlargement of SC, CC, and ISCC in response to systematic steady state increases in SC pressure. At a hydrostatic pressure gradient of 5 mm Hg, only segmental areas of SC lumen were visible, and CC were not visible. With progressive increases of SC pressure, segmental areas of SC lumen expanded and coalesced until by 30 mm Hg the entire length of the canal was distended except the distal portion far from the cannula. Of great interest, the canal lumen dimensions exhibited highly segmental behavior in response to increases in SC pressure of 5 to 10 mm Hg, with not only changes along the canal circumference but also in the plane orthogonal to its length. The CC entrances and their attachment to the adjacent ISCC expanded and new entrances became apparent as SC pressure increased from 10 to 20 mm Hg. As SC pressure further increased to 30 mm Hg, the circumferential channels in ISCC merged to create a fairly uniformly dilated lumen area parallel to SC. The CC entrance openings and ISCC arose in a consistent plane, so a section cutting through their plane of exit creates what appears to be a uniformly thick structure. However, tilting slightly, for example into the YZ plane of Figure 1, will reveal a more complex arrangement.

### 3.2. Morphology with 2D Cross-Sectional Imaging

**Figure 3:**
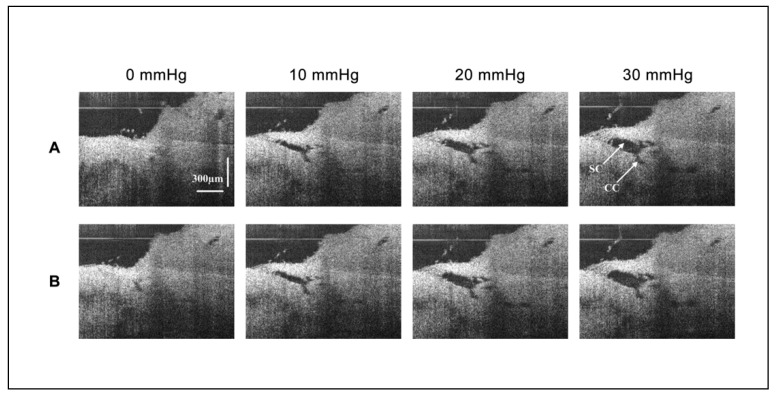
2D-OCT images of the morphological changes of Schlemm’s canal and its surrounding tissues at different steady-state perfusion pressures. (**A**) Presents images of the SC session before bolus. As the pressure increased, the area (instantaneous volume) of SC and CC increased. A structure connected the TM wall of SC to the region of a CC entrance. (**B**) Presents images of the same SC session after bolus. Pressure responses after a large bolus of fluid overexpanded SC and CC. SC pressure-dependent expansion increases. The connection between TM and CC entrance is disrupted. The CC remains modestly dilated, no longer closing at an intraocular pressure of zero mm Hg.

**Figure 4:**
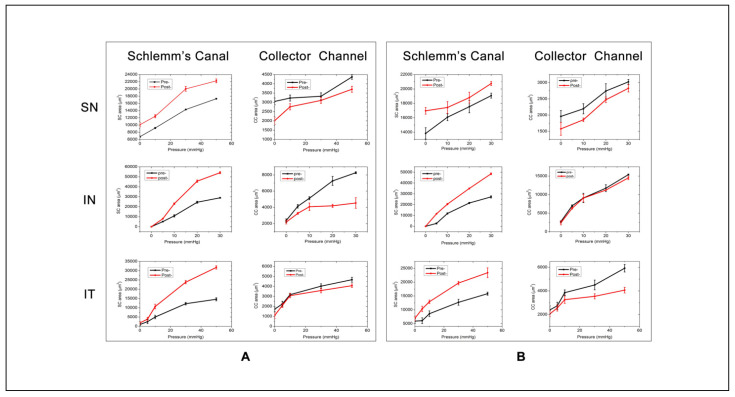
Curves of pressure-dependent changes in Schlemm’s canal and collector channels areas under different perfusion pressures before and after a large fluid bolus. (**A**) The elastance curve of eye #1 and (**B**) eye #2. In each plot, the black line is before and the red line after the fluid bolus. The first row is the superior nasal (SN), the middle the inferior nasal (IN), and the bottom the inferior temporal (IT) quadrant.

The 2D-OCT images show the morphological changes of the same cross-section of SC and its surrounding tissues under different perfusion pressures in Figure 3 (Eye #1 superior temporal quadrant). With the increase of perfusion pressure, SC and its distal CC continued to expand. The valvular structures were identified in the lumen of SC and around the CC entrance. After an aqueous bolus of 30 mL/min and 250 µL, the area of SC increased, while the area of CC decreased compared with their area before fluid bolus perfusion; areas are compared while maintaining identical pressures in SC (Figure 4, Eyes #1 and #2). After the bolus perfusion, the transluminar structure was disrupted and the relative position of the valvular structure around CC shifted.

### 3.3. Volumetric Stress-Strain Curves (Elastance Curves)

**Figure 5:**
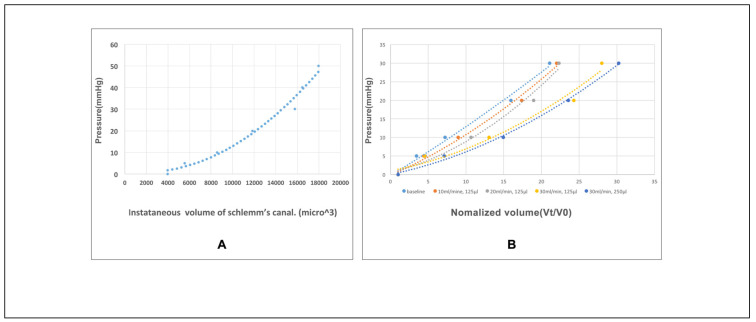
Volumetric stress-strain (elastance) curves of the trabecular meshwork. (**A**) An example of volumetric stress-strain curves of the trabecular meshwork (TM) in the superior temporal (ST) quadrant of the human eye (Eye #3). (**B**) Volumetric stress-strain curves of a 2 mm-long segment of the TM in the ST quadrant at baseline and after incremental fluid infusion in the SC lumen.

Figure 5A displays a volumetric stress-strain curve (elastance curve) representing the relationship between tissue deformation and pressure changes. At each incremental volume increase, the incremental tissue deformation decreases, indicating distention dependent increases in tissue stiffening. The volumetric stress-strain curves of the TM were determined by the relationship between the instantaneous volume of SC and the perfusion pressure in the lumen of SC. The curve characterizes the elastance of the TM by plotting the relationship between tissue deformation and pressure change. Elastance, also the term for tissue stiffness, is the tangent at each location on the curve. As the fluid volume increases, the tissue stiffens, resulting in a nonlinear relationship where a greater incremental pressure rise occurs with each incremental volume increase. In Figure 5B, baseline steady-state elastance curve responses are compared at the infusion rates of 10, 20, and 30 mL/min while holding infusion volume (125 µL), and infusion rates of 30 mL/min with a total volume of 250 µL. The elastance curves move gradually towards the lower right of the plot with incremental infusion bolus increases. It indicates the TM stiffness is changed by the fluid bolus expansion of SC, which mimics the viscoelastic dilation of SC during some MIGS procedures. The degree of TM stiffness change is relevant to the volume and speed of the infusion fluid.

**Figure 6:**
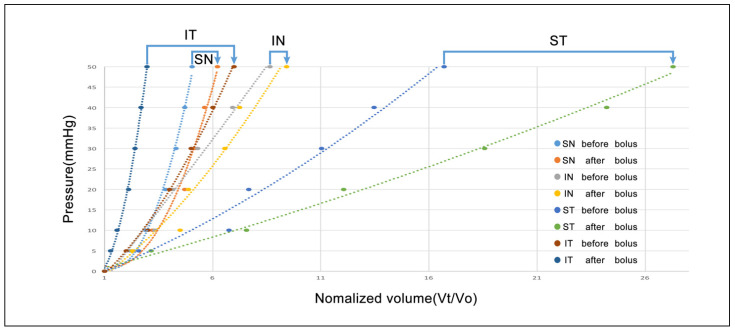
Changes of elastance curves of the trabecular meshwork induced by a fluid bolus. Blue arrows indicate the before and after curves for the same quadrant. ST: superior temporal; IT: inferior temporal; SN: superior nasal; and IN: inferior nasal.

The elastance of TM is variable among the quadrants. After the infusion of fluid bolus of 30 mL/min and 250 µL, the TM stiffness all shifted to the bottom right of the plot, indicating the decrease of TM stiffness. The degree of TM stiffness change induced by the fluid expansion of SC is dependent on the initial status of TM (Figure 6, eye #3).

## 4. Discussion

In this pilot study, our approach circumvents the problem posed by viscoelastic SC dilation, which does not permit the study of dynamic motion. Instead, we cannulate and dilate SC using an aqueous fluid that permits varying control of same sample SC pressures. While systematically controlling pressure gradients, we simultaneously monitor the configuration of TM, SC, and ISCC with HR-OCT. Our technique permits the same-sample comparative assessment of TM biomechanical properties, which avoids the limitation of variation between tissue samples. The TM configuration determines SC dimensions. As the ventricular volume change during the cardiac cycle represents the function of myocardium, we use SC dimension changes as a means of assessing TM behavior. In this study, we quantitate stress-strain relationships (elastance) determined by TM responses to changes in pressure.

Our HR-OCT study permitted us to explore morphology relationships of SC, CC, and ISCC using 3D volumetric imaging. We were able to examine both the stress-strain relationships of the TM and CC and the synchrony of changing dimensions of the tissues. In addition, our experimental protocol permitted us to study changes in biomechanical properties of the tissues following simulation of SC dilation with MIGS-like approaches.

The 3D volumetric information provided by HR-OCT permits us to visually examine the relationship between the TM, CC, and ISCC through dynamic rotation of the tissues along the XY, XZ, and YZ axis. The dimensions of SC, CC, and ISCC decrease, moving from the proximal areas adjacent to the cannula to the more distal circumference of SC. The decrease in SC dimensions with distance from the site of infusion is consistent with findings from histologic studies following viscoelastic injections into SC [20].

The views perpendicular to CC exit sites confirm that CCs exit from SC in a uniform, rather narrow plane and join circumferentially oriented ISCC. Through-cornea viewing of microvascular casts permits viewing CC perpendicular to their sites of exit from SC and is consistent with the findings of this study. The findings are also consistent with those from SEM and micro-CT studies [17,18,19].

HR-OCT 3D morphology reconstructions demonstrate the ability of the TM and tissues surrounding CC to change shape (Figure 1). Without pressure in SC, the lumen is small and connections between regions of the lumen are patchy [16,22,23]. As SC pressure increases, the disconnected regions of the lumen of SC coalesce, creating a continuous structure. With the increasing SC pressure, CC lumen dimensions increase. With further pressure increases, the ISCC fill and segmental areas also coalesce. The findings indicate that in the absence of pressure, elastance properties of the tissues cause them to recoil, reducing the dimensions of SC, CC, and ISCC. Elastance properties of the tissues permit the TM, as well as the tissues surrounding CC and ISCC to expand, increasing available areas for aqueous flow [24].

The evidence from steady state 2D configuration imaging confirms findings with 3D imaging, showing that the TM and tissue surrounding the CC and ISCC are not distended at 0 mmHg. The lumen dimensions of CC and ISCC become progressively more visible as SC pressure increases, demonstrating the elastance properties of the tissue surrounding their volumes. Connections between the TM and CC entrances are also visible and are especially obvious when interrogating the 2D volumes by increasing SC pressure gradients during experiments. The changes in CC dimensions provide increasing space through which aqueous can flow from SC into ISCC.

A reverse finite element model (FEM) studied the bulk motion of normal and glaucomatous TM lamellae under tensile loading conditions. The FEM model used experimental control of transtrabecular hydrostatic pressure gradients such as those described in the current study. The elastic modulus of normal human the TM estimated by inverse FEM was 70 ± 20 kPa (mean 6 SD), whereas glaucomatous human TM was slightly stiffer (98 ± 19 kPa) reaching borderline significance with a *p*-value of 0.051. Outflow facility was significantly correlated with TM stiffness estimated by FEM in six human eyes (*p* = 0.018) [25].

As in the FEM study, our HR-OCT system measures the bulk motion of the TM tissues represented by the motion of the trabecular lamellae. The SC inner wall endothelium dimensions and motion responses are too small to be resolved with our current technology. By examining the relationship between an increase in SC area and the resulting relationship between pressure and tissue deformation we can characterize the TM tissue elastance.

Elastance is the ability of a hollow organ to recoil toward its original dimensions on the removal of a distending force and represents the ability to store and release elastic energy [26]. The stored energy associated with an increasing volume distributes between the elastic energy of tissue deformation and pressure energy. The TM maintains its position against the force of IOP through its elastance properties that determine how far the TM distends into SC when IOP rises. Elastance properties also determine the amplitude of TM excursions in response to pulse oscillations. The measurements we obtain with elastance curves allow us to explore the complex relationship of the distribution of energy associated with volume-dependent tissue deformation and pressure.

We find an increase in SC lumen area as SC pressure increases indicative of TM tissue deformation with the accrual of elastic energy (Figure 5A). Lumen area configuration depends on the biomechanical properties of the surrounding tissues. We develop volumetric stress-strain curves (elastance curves) using volume as the dependent variable. However, we add rapidity of volume change as a second experimentally controlled variable (Figure 5B). We find synchronous SC and CC lumen dimensions changes (Figure 4) indicative of synchrony of the TM motion and the tissue surrounding CCs. The correlated movement of the TM and CC entrance tissues is consistent with the findings of prior studies [15].

Our pilot study has clinical relevance because it is the first to use OCT to demonstrate changes in biomechanical properties of the outflow system in response to MIGS-like procedures that dilate SC (Figure 4). To regulate IOP, elastance properties of the outflow system must be kept in a narrow range. Either too high or too low elastance will result in loss of TM motion, a loss thought to be a hallmark of the glaucoma process [4,13].

Increased elastance/stiffness will prevent the motion necessary for the TM to alter outflow channel dimensions in response to IOP changes and reduce the ability to respond to the ocular pulse. Compliance is the inverse of elastance, so reduced elastance results in increased compliance. High compliance alters the ability of the TM to withstand the force of IOP, and herniation and apposition to SC external wall can result [27]. In the absence of normal elastance, the TM will also not undergo appropriate excursions in response to the ocular pulse or pressure transients associated with blinking and eye movement.

Our steady-state experiments demonstrate that SC areas increase, but CC areas decrease compared with their original dimensions at an individual level of perfusion pressure in response to experimental SC dilation that disrupts TM-CC connections. MIGS-like procedures that dilate SC were thus found to alter the biomechanical properties of both the TM and tissue surround CC in our experiments. Our study as well as prior viscoelastic studies demonstrate disruption of valvular structure traversing the SC lumen and their attachments at CC entrances. We hypothesize that disruption of connections between the TM and CC entrances reduces the ability of the CC entrances to move in response to pressure-dependent TM motion [5].

Volumetric stress-strain (elastance) curves explore the effects of volume increases on biomechanics before and after expansion of SC with a fluid bolus in all four quadrants of an eye (Figure 6). The elastance curve varied markedly between quadrants, consistent with segmental and regional variability. However, in each quadrant, the elastance curves shifted toward the right and steepened, indicating that biomechanical properties of the TM change with SC overexpansion. Whether the altered elastance will be beneficial is dependent on the interplay between the initial enlargement of the outflow channels and disruption of connections between the TM and CC entrances. Disruption of TM-CC entrances may reduce the ability of the recoiling TM to maintain appropriate tensions to hold CC entrances open.

## 5. Conclusions

Our study explores morphologic relationships and biomechanical properties of the outflow system in ex vivo human eyes using our HR-OCT platform. Using 3D morphometric imaging, we identify the ability of SC, CC, and ISCC to undergo synchronous changes in shape in response to changes in SC pressure. We find the CCs exit SC in a relatively uniform plane to then enter the circumferentially oriented ISCC.

Volumetric stress-strain studies demonstrate the ability to develop elastance curves. The curves identify synchronous SC and CC dimension increases with increasing SC pressures. Assessment of these biomechanical properties can improve our understanding of normal mechanisms of IOP control and the abnormalities in glaucoma.

Our study finds that MIGS-like overexpansion of SC causes alterations in elastance properties of the TM and tissues surrounding the CC. The canal overexpansion causes changes in the biomechanical responses of the TM, resulting in a pressure-induced increase of the motion that determines SC dimensions. At the same time, there is a decrease in the motion response of the tissues surrounding CC, resulting in reduced lumen dimension changes. The reduced CC response may result from the disruption of attachments between the TM and the attachments that control CC entrance dimensions.
